# Design and pharmacodynamics of recombinant NZ2114 histidine mutants with improved activity against methicillin-resistant *Staphylococcus aureus*

**DOI:** 10.1186/s13568-017-0345-x

**Published:** 2017-02-22

**Authors:** Huixian Chen, Ruoyu Mao, Da Teng, Xiumin Wang, Ya Hao, Xingjun Feng, Jianhua Wang

**Affiliations:** 1 Institute of Animal Nutrition, Northeast Agricultural University, Harbin, 150030 China; 2 Key Laboratory of Feed Biotechnology, Ministry of Agriculture, Beijing, 100081 China; 3 Gene Engineering Laboratory, Feed Research Institute, Chinese Academy of Agricultural Sciences, Beijing, 100081 China

**Keywords:** Antimicrobial peptide, NZ2114, Pharmacodynamics, *Staphylococcus aureus*

## Abstract

NZ2114 is a promising candidate for therapeutic application owing to its potent activity to *Staphylococcus aureus*. Our objective was to identify NZ2114 derivatives with improved activity through substitution of His16 and His18 with residues Arginine and Lysine. Eight mutants were designed and expressed in *Pichia pastoris* X-33 via pPICZαA. Five of them exhibited strong antimicrobial activity against *S. aureus* at low minimal inhibitory concentrations (MICs) of 0.057–0.454 μM. Among them, H1, H2, and H3 showed ideal pharmacodynamic effects on methicillin-resistant *S. aureus* ATCC43300.  The total protein level of H1, H2, and H3 reached 1.70, 1.77 and 1.54 g/l at 120 h of induction in the 5-l fermenter, respectively. They killed over 99.9% of pathogens within 1.5 h at 2× and 4× MIC. The post antibiotic effect of H1, H2 and H3 to *S. aureus* ATCC43300 was 2.94, 1.75 and 1.55 h at 2× MIC, which was similar with their original peptide NZ2114 (1.43 h) and vancomycin (1.72 h). The fractional inhibitory concentration index (FICI) indicated indifferent effects between H1, H2, H3 and vancomycin, ampicillin, rifampicin. Additionally, they had low hemolysis and high stability in different environments (temperature, pH, proteases, and saline ions). All results indicate that H1, H2, and H3 can be produced in large-scale and have potential as therapeutic drugs against MRSA.

**Electronic supplementary material:**

The online version of this article (doi:10.1186/s13568-017-0345-x) contains supplementary material, which is available to authorized users.

## Introduction

Methicillin-resistant *Staphylococcus aureus* (MRSA) is one of the most common antimicrobial-resistant pathogens causing invasive infections (Dantes et al. 2013), which can produce a series of toxins and shows frequent and sometimes multi-drug resistance to antimicrobials (Stefani et al. 2012). According to the previous report, 25–35% of healthy human individuals carried *S. aureus* on the skin or mucous membranes (Wertheim et al. 2005). This means that up to two billion individuals may currently carried *S. aureus* worldwide, and conservative estimates based on Dutch and US prevalence data predicted that 2–53 million people carried MRSA (Grundmann et al. 2010). MRSA infections can affect more than 150,000 patients annually in the European Union (EU), resulting in extra in-hospital costs of EUR 380 million for EU healthcare systems (Köck et al. 2010). In another report, it is estimated that MRSA caused 171,200 healthcare-associated infections (HAIs) in Europe each year, which equals to 44% of all HAIs. MRSA also caused 5400 extra deaths and over a million extra days of hospitalisation associated with the infection (Gould et al. 2012).

The guidelines issued by Infectious Disease Society of America in 2011 recommend vancomycin, daptomycin, linezolid or clindamycin for MRSA infection (Liu et al. 2011). Although MRSA can be effectively treated by some antibiotics, sometimes their MIC values increased. Data from the tigecycline evaluation and surveillance trial showed that proportion of MRSA with MICs (vancomycin) ≥2 mg/l increased from 5.6% in 2004 to 11.1% in 2009 (*P* < 0.001) (Hawser et al. 2011).

Antimicrobial peptides (AMPs) are potent drugs known for their broad-spectrum of antimicrobial properties and particularly against antibiotic-resistant bacteria (Brogden and Brogden 2011). However, some flaws hamper the clinical usage of AMPs, such as toxicity to normal mammalian cells and the lack of a cost-effective way of commercial-scale production (Cao et al. 2015). Plectasin is a fungal defensin from *Pseudoplectania nigrella* and is especially active against Gram-positive bacteria such as *S. aureus* (MIC_50_: 16 μg/ml for methicillin-sensitive strains and 32 μg/ml for resistant strains) and *Streptococcus pneumoniae* (MIC_50_: 1 μg/ml for both penicillin-sensitive and resistant strains) by binding with the pyrophosphate moiety of lipid II, the essential precursor of the cell wall (Mygind et al. 2005; Ostergaard et al. 2009; Schneider et al. 2010). Peptide NZ2114 is a novel variant of plectasin (D9N, M13L, Q14R) that is significantly more potent than parental peptide (MIC_50_: 2 μg/ml for *S. aureus* and 0.25 μg/ml for *S. pneumoniae*) (Ostergaard et al. 2009; Zhang et al. 2014; Andes et al. 2009; Xiong et al. 2011). It also had long postantibiotic effect (PAE) (Zhang et al. 2014) and synergistic in combination with the conventionl antibiotics such as teicoplanin, moenomycin, and dalbavancin (Breidenstein et al. 2015). It had potent activities against *S. aureus* in rabbit meningitis, murine peritonitis, and thigh infection models (Ostergaard et al. 2009; Andes et al. 2009; Xiong et al. 2011). Additionally, NZ2114 showed low or no cell toxicities, long-lasting serum stability and in vivo half-life (Brinch et al. 2010).

However, two histidine (His) residues exist in the sequence of NZ2114, which have a pKa of approximately 6.0 and are largely unprotonated and uncharged at physiological conditions (Kashiwada et al. 2016). It was found that some AMPs rich in His exhibited higher activity at low pH, when the histidine was positively charged, as compared with neutral pH. For example, LAH4 weakly disrupted the membrane at physiological conditions. The cell lysis activities of peptides LL-1a and LL-1c decreased up to four times as the solution pHs at 7.4 (Mason et al. 2006). The arginine residues were previously suggested to be important for the antimicrobial activity of AMPs (Tu et al. 2009) and also to potentiate the internalization of peptides (Hansen et al. 2008). Arginine residues having a more dispersed positive charge on their side chain guanidinium group, have been reported to enhance the electrostatic interactions between peptides and anionic lipids (Strandberg et al. 2002). Veiga et al. (2012) demonstrated that the self-assembling β-hairpin peptides, having a high content of arginine, were extremely effective to killing both gram-positive and gram-negative bacteria, including multi-drug resistant strains. Silva et al. (2014) found that arginine residues were crucial for the display of antimycobacterial activity. On the other side, the positive charge of lysine residues were considered to be critical for interacting with the anionic interface as a result of initial electrostatic interaction (Koba et al. 2009). It showed that four positively charged lysine residues concentrated on the hydrophilic face of HPA3NT3-analog peptides, improved the amphipathic structure, and resulted in decrease of hemolysis (Gopal et al. 2009). The large loss of antimicrobial activity was also found when the C-terminal arginine and lysine residues of CL(14–25) was replaced with alanine residues (Taniguchi et al. 2014).

Therefore, to enhance the antimicrobial activity of NZ2114, replacement of the histidine at the 16th and 18th positions by arginine and lysine was performed. Eight mutants were designed and the antibacterial activities were evaluated. The peptides with excellent antibacterial property were chosen to test their pharmacodynamics, post antibiotic effect, synergy, hemolytic activity, and stability against *S. aureus* ATCC43300 (MRSA).

## Materials and methods

### Strains, plasmid and reagents

*Escherichia coli* DH5α, *P. pastoris* X-33 and pPICZαA vector were purchased from Invitrogen (Beijing, China). The test strains for the antimicrobial activity assays and their sources are listed in Additional file 1: Table S1. Vancomycin, ampicillin, rifampicin and ciprofloxacin were purchased from the China Institute of Veterinary Drug Control. NZ2114 was prepared in our laboratory (Zhang et al. 2014). Restriction enzymes and T4 DNA ligase were purchased from New England Biolabs (NEB, Beijing, China). The kits for plasmid extraction and DNA purification were purchased from Tiangen (Beijing, China). Other chemical reagents were analytical grade.

### Peptide design

The main structure of NZ2114 was kept unchangable to maintain the antimicrobial activity. Additionally, the histidine residues in position 16 and 18 were mutated into arginine or lysine. As results, eight derived peptides which H16 and H18 were replaced by arginine or lysine were generated. The amino acid sequences and properties of the parental peptide and designed peptides were calculated by bioinformatics programs, including antimicrobial peptide calculator and predictor (http://aps.unmc.edu/AP/prediction/prediction_main.php) and ProtParam (ExPASy Proteomics Server: http://www.expasy.org/tools/protparam.html). All of the parameters are summarized in Table 1.

**Table 1:** Amino acid sequences and physicochemical properties of H1–H8

Name	Sequence	Molecular weight (Da)	PI	Charge	GRAVY	Instability index:	Boman index (kcal/mol)
NZ2114	GFGCNGPWNEDDLRCHNHCKSIKGYKGGYCAKGGFVCKCY	4417.0	8.62	+ 3	−0.672	25.49	1.52
NZ16K (H1)	GFGCNGPWNEDDLRCKNHCKSIKGYKGGYCAKGGFVCKCY	4408.0	8.84	+4	−0.690	11.42	1.54
NZ16R (H2)	GFGCNGPWNEDDLRCRNHCKSIKGYKGGYCAKGGFVCKCY	4436.0	8.86	+4	−0.705	14.51	1.77
NZ18K(H3)	GFGCNGPWNEDDLRCHNKCKSIKGYKGGYCAKGGFVCKCY	4408.0	8.84	+4	−0.690	31.41	1.54
NZ18R (H4)	GFGCNGPWNEDDLRCHNRCKSIKGYKGGYCAKGGFVCKCY	4436.0	8.86	+4	−0.705	25.49	1.77
NZ16K18K (H5)	GFGCNGPWNEDDLRCKNKCKSIKGYKGGYCAKGGFVCKCY	4399.0	9.02	+5	−0.708	17.34	1.56
NZ16K18R(H6)	GFGCNGPWNEDDLRCKNRCKSIKGYKGGYCAKGGFVCKCY	4427.1	9.04	+5	−0.722	11.42	1.79
NZ16R18K (H7)	GFGCNGPWNEDDLRCRNKCKSIKGYKGGYCAKGGFVCKCY	4427.1	9.04	+5	−0.722	20.43	1.79
NZ16R18R (H8)	GFGCNGPWNEDDLRCRNRCKSIKGYKGGYCAKGGFVCKCY	4455.1	9.06	+5	−0.738	4.51	2.03

### Construction of recombinant plasmid pPICH1–pPICH8

The codon-optimized gene sequences of H1–H8 (Additional file 1: Table S2) were designed by the Reverse Translate Tool (www.bioinformatics.org/sms2/rev_trans.html), according to the preferential codon usage of *P. pastoris* (www.kazusa.or.jp/codon/). To ensure the integrity of the sequences in the expression process, the expression cassette included an *Xho*I recognition site, a *P. pastoris* Kex2 protease cleavage site, the H1–H8 genes, two stop codons, and an *Xba*I recognition site. The DNA sequences and pPICZαA vector were digested by *Xho*I and *Xba*I, gel-purified, and ligated together by T4 DNA ligase. The recombinant plasmids pPICH1–pPICH8 were transformed into *E. coli* DH5α, and positive cells were selected. The H1–H8 gene sequences were confirmed by DNA sequencing using the following two primers.

Primer 5′AOX1: 5′-GACTGGTTCCAATTGACAAGC-3′

Primer 3′AOX1: 5′-GCAAATGGCATTCTGACATCC-3′

F1: 5′-CCGCTCGAGAAGAGAGGTTT-3′

R1: 5′-GCTCTAGATTATTAGTAACAC-3′

### Transformation and selection of positive transformants

The pPICH1–pPICH8 were linearized with *Pme*I and then transformed into the competent *P. pastoris* X-33 cells by electroporation following the Invitrogen’s instructions. The pPICZαA vector was also linearized and transformed into *P. pastoris* X-33 cells as a negative control. All zeocin-resistant colonies were selected in YPDS plates (10 g/l yeast extract, 20 g/l peptone, 20 g/l glucose, 182.2 g/l sorbitol, 20 g/l agar, and 100 μg/ml zeocin).

### Expression of H1–H8 in *P. pastoris* in 48-well plates

A single colony of positive *P. pastoris* transformants was cultured at 29 °C (250 rpm) in 48-well plates containing 500 µl BMGY medium (10 g/l yeast extract, 20 g/l peptone, 10 ml/l glycerol, 13.4 g/l yeast nitrogen base, 400 µg/l biotin, and 100 ml/l 1 M potassium phosphate, pH 6.0). After 24 h, methanol (100%) was added each well to a final concentration of 0.5% (v/v), and the temperature was adjusted to 28 °C. Then methanol was repeatedly added every 24 h during the 96 h induction time. The supernatant was collected by centrifugation at 10,000×*g* for 10 min and stored at −20 °C. The expression conditions of H1–H8 were determined by the inhibition zone assay against *S. aureus* ATCC25923 and Tricine-sodium dodecyl sulfate polyacrylamide gel electrophoresis (Tricine-SDS-PAGE) (Zhang et al. 2014; Schägger 2006). Then the positive transformants with high inhibitory effect were selected to express in shake flasks (1-l shake flask containing 200-ml BMGY medium) using the same method.

### Purification and identification of H1, H2, H3, H6, H8

The supernatant of 1-l shake flasks of H1, H2, H3, H6, and H8 was applied onto an SP Sepharose FF cation-exchange column (GE Healthcare, UK) pre-equilibrated with binding buffer (20 mM sodium phosphate buffer, pH 6.7). H1, H2, H3, H6, and H8 were eluted from the column with elution buffer (20 mM sodium phosphate buffer, 600 mM NaCl, pH 6.7) at a rate of 6 ml/min, and the eluent of corresponding elution peaks were collected. The eluent were analyzed by Tricine-SDS-PAGE and confirmed by MALDI-TOF MS at the Laboratory of Proteomics, Institute of Biophysics, Chinese Academy of Sciences according to the previously reported method (Zhang et al. 2014).

### Antimicrobial activity assays of H1, H2, H3, H6, and H8 in vitro

The peptide solutions were diluted twofold with the range of final concentrations were 0.015–32 µg/ml (0.003–7.273 µM) for purified H1, H2, H3, H6, and H8. The test strains were grown in MHB medium at 37 °C to an OD_600_ of 0.4 and diluted to 1 × 10^5^ CFU/ml. The 10 µl peptide and 90 µl cell suspension were added into each well. All assays were performed in triplicate. The antimicrobial activity of NZ2114 and vancomycin were tested as positive controls. The plates were incubated at 37 °C for 18–24 h. MIC was defined as the lowest peptide concentration of ones at which there was no visible growth (Tian et al. 2009).

### Expression of H1, H2, H3 in *P. pastoris* in high-density cultivation in fermentors

According to the results of the antimicrobial activity assays, H1, H2, and H3 were chosen to be expressed in the 5-l fermentor. A single colony of H1, H2, H3 was incubated in shaking flasks with 10 ml YPD medium at 30 °C (250 rpm). Overnight cultures were inoculated into 200 ml YPD medium and cultivated at 30 °C (250 rpm) to an OD_600_ of 5.0 and then transferred into a 5-l fermentor (BIOSTAT®B plus, Sartorius Stedim Biotech) containing 2 l basal salts medium (50 g/l NH_4_H_2_PO_4_, 20 g/l K_2_SO_4_, 15 g/l MgSO_4_·7H_2_O, 6 g/l KH_2_PO_4_, 0.4 g/l CaSO_4_, 1.5 g/l KOH, 45 g/l glucose, and 4.8‰ PMT1). The pH was controlled at 5.5 using H_3_PO_4_ and NH_4_OH, and the temperature was maintained at 29 °C. When the glucose was exhausted, methanol was supplied from 1 to 7 ml/l/h during the first 6 h. Then methanol was supplied to maintain a relative dissolved oxygen (DO) content between 20 and 40% under the speed of 6–8 ml/l/h (Bai et al. 2010). The fermentation liquid was collected every 24 h to quantify the cell wet weight and the total protein level which was assayed by a Bradford protein assay kit (Tiangen Biotech, Beijing, China). The expression of H1, H2, and H3 was determined by Tricine-SDS-PAGE. The supernatant was purified in the same way as the 1-l shake flasks.

### Time-kill curve assay

Bacterial culture (*S. aureus* ATCC43300) was diluted to 1 × 10^5^ CFU/ml and H1, H2, and H3 were added. The final concentration of H1, H2, and H3 was 1× , 2× and 4× MIC, respectively. The mixture was cultivated at 250 rpm, 37 °C. The 100-µl samples were taken from each flask at 0, 2, 4, 6, 8, 12 and 24 h of incubation, and serial dilutions of samples were plated to count visible colonies. Vancomycin was tested in the same way as a positive control and the culture without antimicrobial agent as a negative control (Xiong et al. 2011). All experiments were performed in triplicate.

### The postantibiotic effect of H1, H2, H3 against *S. aureus*

The 100 µl peptides were added into tubes containing 900 µl bacterial cultures (1 × 10^8^ CFU/ml) to make their final peptide concentration to 1× , and 2× MIC, respectively, and the mixture was cultured at 37 °C for 2 h. Tubes with 2× MIC vancomycin, 2× MIC NZ2114, and without antimicrobial agent were used as controls. After 2 h, the drug was removed by diluting 1:10^3^ into the MHB and incubated at 37 °C, 250 rpm. The sample was taken to plate counting every 1 h until the bacterial cultures become turbid. The PAE was calculated by the following formula: PAE = T − C, where T is the time needed for the count of CFU in the test culture to increase 1 log_10_ (10-fold) above the 0 h and C is the time needed for the count of CFU in the untreated control culture to increase 1 log_10_ above the 0 h (Giguère et al. 2012).

### Synergism assays of H1, H2, H3 with conventional antibiotics

The MIC values of four antibiotics (vancomycin, ampicillin, rifampicin and ciprofloxacin) to *S. aureus* ATCC43300 were tested as the MIC assay described above. The peptide solutions and antibiotics were diluted twofold with the final concentrations ranging from 1/16 to 8× MIC, and added into 96-well plates in a checkerboard fashion (White et al. 1996). The results of combination were evaluated by calculating the fractional inhibitory concentration index (FICI) of each combination. FIC of H1, H2, H3=MIC of H1, H2, H3, respectively, in combination with antibiotic/MIC of H1, H2, H3 alone; FIC of antibiotics=MIC of antibiotic in combination with peptide/MIC of antibiotic alone; FICI = FIC of H1, H2, H3+ antibiotic, respectively. The result of interaction between two antimicrobial drugs was determined according to: FICI ≤ 0.5 refers to synergy, 0.5 < FICI ≤ 1 refers to additivity, 1 < FICI ≤ 4 refers to indifference, and FICI > 4 is defined as antagonism (Tsuji and Rybak 2006).

### Hemolytic assay

The mice blood cells were washed three times in stroke-physiological saline solution (0.9% NaCl) and centrifuged at 4 °C, 2000 rpm for 5 min. A 50-μl cell was diluted to 8% (v/v), added into 96-well plates, and mixed with 50-μl peptide to the final concentrations ranging from 0.23 to 23.09 μM (1–128 μg/ml). The plates were incubated at 37 °C for 1 h, and centrifuged at 4 °C, 5000 rpm for 5 min. The absorbance of supernatants was measured at 540 nm, and 0 and 100% hemolysis was measured by 0.9% NaCl and 0.1% Triton X-100, respectively. The hemolysis percentages were calculated by the following equation: [(Abs540 nm in H1, H2, H3 solution − Abs540 nm in 0.9% NaCl)/(Abs540 nm in 0.1% Triton X-100 − Abs540 nm in 0.9% NaCl)] ×100% (Jiao et al. 2015).

### Circular dichroism (CD) of H1, H2, H3

CD spectroscopy analysis of H1, H2, H3 [5.68 µM (25 µg/ml) in ddH_2_O, 20 mM SDS, 50% TFE solution was carried out in a MOS-450 spectropolarimeter (Bio-Logic, Grenoble)]. The samples were loaded into a 1 mm cell path at room temperature and the spectra were recorded from 195 to 245 nm three times.

### Effect of pH, temperature, NaCl concentration and proteinase on the activity of H1, H2, H3

The effect of pH, temperature, NaCl concentration and proteinase on the activity of peptides was evaluated. Peptides were diluted twofold, then adjusted to a pH range from 2.0 to 10.0 and incubated at 37 °C for 4 h. The thermal stability of purified peptides was determined after 1-h incubation of peptides at 4, 20, 40, 60, 80 and 100 °C in deionized water, respectively. Aliquots of peptides were incubated in various proteinases solutions [pepsin (pH 2.0), trypsin (pH 8.0), proteinase K (pH 7.0)] at a ratio of 1:10, w/w (proteinase: peptide) at 37 °C for 3 h. For the ion stability, peptides were incubated in 50, 100, 200, 300, 400, 500 mM sodium chloride solutions, respectively. Other methods and conditions were prepared as the MIC assay described above (Li et al. 2015; Qu et al. 2016).

## Results

### Peptides design

The net charge of derived peptides with one site mutants (H1, H2, H3 and H4) increased from +3 to +4, and the two sites mutants was +5 (H5, H6, H7 and H8). Due to the higher hydrophily of arginine and lysine, the grand average of hydropathicity (GRAVY) slightly decreased for one site mutants (from −0.672 to −0.690 for H1 and H3, and to −0.705 for H2 and H4, respectively), and it further decreased for two sites mutants (−0.708 for H5, −0.722 for H6 and H7, and −0.738 for H8, respectively). The related high hydrophilicity may contribute to the surface contact of pathogens and peptides. The Boman index indicates the binding activity of drugs to protein, the large value may induce high negative side effects. Generally, it was considered that the value was acceptable as 1–3 kcal/mol (Weistroffer 2007). The Boman index of derived peptides was all between 1.54 and 2.03 kcal/mol, indicating they may have little negative side effects as novel antimicrobial agents (Table 1).

### Expression of H1–H8 in *Pichia pastoris* in 48-well plates

Thirty-six positive transformants of each peptide were screened by inhibition zone against *S. aureus* ATCC25923. The transformants showed various antimicrobial activity except H4 (date not shown). According to the diameter of inhibition zone, one transformant of each peptide was selected to analyze the expression condition by Tricine-SDS–PAGE. As shown in Additional file 1: Figure S1, there were no visible bands in the lane of H4, H5, H7, while H1, H2, H3, H6, and H8 displayed obviously bands, so they were chosen to the following assays.

### Purification and identification of H1, H2, H3, H6, and H8

**Fig. 1:**
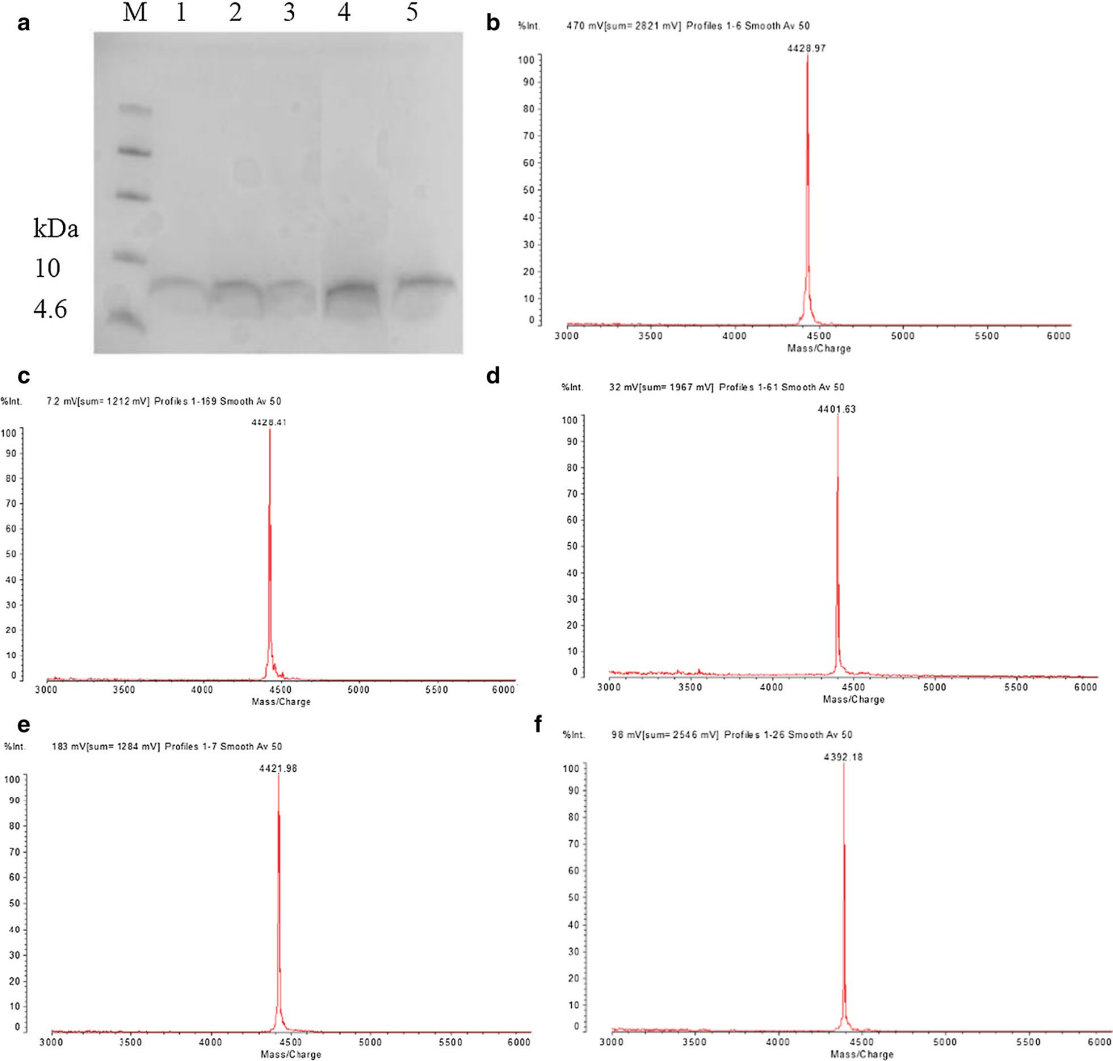
Purification and identification of H1, H2, H3, H6, and H8. **a** Tricine-SDS-PAGE analysis of H1, H2, H3, H6, and H8 in fermentation supernatants of 1-l shake flasks. Lane M a total of 6 μl of protein molecular weight marker. Lane 1–5, 10 μl of purified H1, H2, H3, H6, and H8, respectively. **b**–**f** MALDI-TOF MS analysis of the purified H1, H2, H3, H6, and H8 fermentation supernatants in 1-l shake flasks

The supernatants of H1, H2, H3, H6, and H8 in 1-l shake flasks were purified. H1, H2, H3, H6, and H8 were detected by Tricine-SDS-PAGE from the eluent containing 20 mM sodium phosphate buffer, 600 mM NaCl, pH 6.7 (Fig. 1a). Target bands around 4 kDa were detected, and there were no other bands (Fig. 1a). MALDI-TOF MS analysis indicated that only a target peak of 4428.97, 4428.41, 4401.63, 4421.98, and 4392.18 Da from purified peptides (Fig. 1b–f) were detected, which were consistent with their theoretical value of 4428, 4428, 4402, 4421, and 4393 Da, respectively.

### Antimicrobial activity assays of H1, H2, H3, H6, and H8 in vitro

H1, H2, H3, H6, and H8 showed a potent antimicrobial activity against Gram-positive bacteria, including three *S. aureus* strains (MIC: 0.057–0.454 μM), three *Streptococcus suis* strains (MIC: 0.007–0.057 μM), and two *S. pneumonia* strains (MIC: 0.227–1.818 μM) (Table 2). Due to the higher charge and hydrophily of arginine and lysine compared to histidine, their activity (MIC: 0.057–0.454 μM) against *S. aureus* ATCC43300 (MRSA) was improved two to sixteen times than NZ2114 (0.909 μM) and two to twelve times than vancomycin (0.714 μM). In addition, the activity of H1, H2, and H3 against Methicillin-susceptible *Staphylococcus aureus* (MSSA ATCC25923, and ATCC6538) was improved twice than NZ2114. Meanwhile, the too high charge and hydrophily did not result in the improved activity of H6 and H8 against both MRSA (ATCC43300) and MSSA (ATCC6538). All of them could not inhibit Gram-negative pathogens (Table 2). Due to the excellent activity of H1, H2, and H3 against *S. aureus*, they were selected for the further pharmacodynamics study.

**Table 2:** MIC assays of H1, H2, H3, H6, H8, NZ2114, and vancomycin against G^+^ and G^−^ pathogens

Strains	MIC (μM)						
	H1	H2	H3	H6	H8	NZ	Van
Gram-positive bacteria							
*S. aureus* ATCC25923	0.014	0.028	0.014	0.028	0.028	0.028^a^	0.172
*S. aureus* ATCC43300	0.057	0.114	0.057	0.114	0.454	0.909^a^	0.714
*S. aureus* ATCC6538	0.057	0.057	0.114	0.114	0.227	0.114^a^	1.428
*S. suis* CVCC3309	0.014	0.028	0.014	0.028	0.028	0.028	0.172^b^
*S. suis* CVCC3928	0.007	0.014	0.028	0.014	0.057	0.028	0.172^b^
*S. suis* CVCC606	0.007	0.028	0.028	0.014	0.028	0.028	0.172^b^
*S. pneumoniae* CVCC1.8722	0.227	0.227	0.454	0.454	1.818	0.454	NT
*S. pneumoniae* CVCC2350	0.227	0.227	0.227	0.454	1.818	0.909	NT
Gram-negative bacteria							
*S. enteritidis* CMCC50336	>7.273	>7.273	>7.273	>7.273	>7.273	>7.273	NT
*S. typhimurium* ATCC14028	>7.273	>7.273	>7.273	>7.273	>7.273	>7.273	NT
*S. choleraesuis* CVCC503	>7.273	>7.273	>7.273	>7.273	>7.273	>7.273	NT
*S. pullorum* CVCC1789	>7.273	>7.273	>7.273	>7.273	>7.273	>7.273	NT
*E. coli* CVCC195	>7.273	>7.273	>7.273	>7.273	>7.273	>7.273	NT
*E. coli* CICC21530	>7.273	>7.273	>7.273	>7.273	>7.273	>7.273	NT

*NZ* NZ2114, *Van* vancomycin, *NT* no test

^a^The data are from previous results (Zhang et al. 2014)

^b^The data are from previous results (Jiao et al. 2015)

### Expression of H1, H2, H3 in *P. pastoris* in high-density cultivation in fermenters

**Fig. 2:**
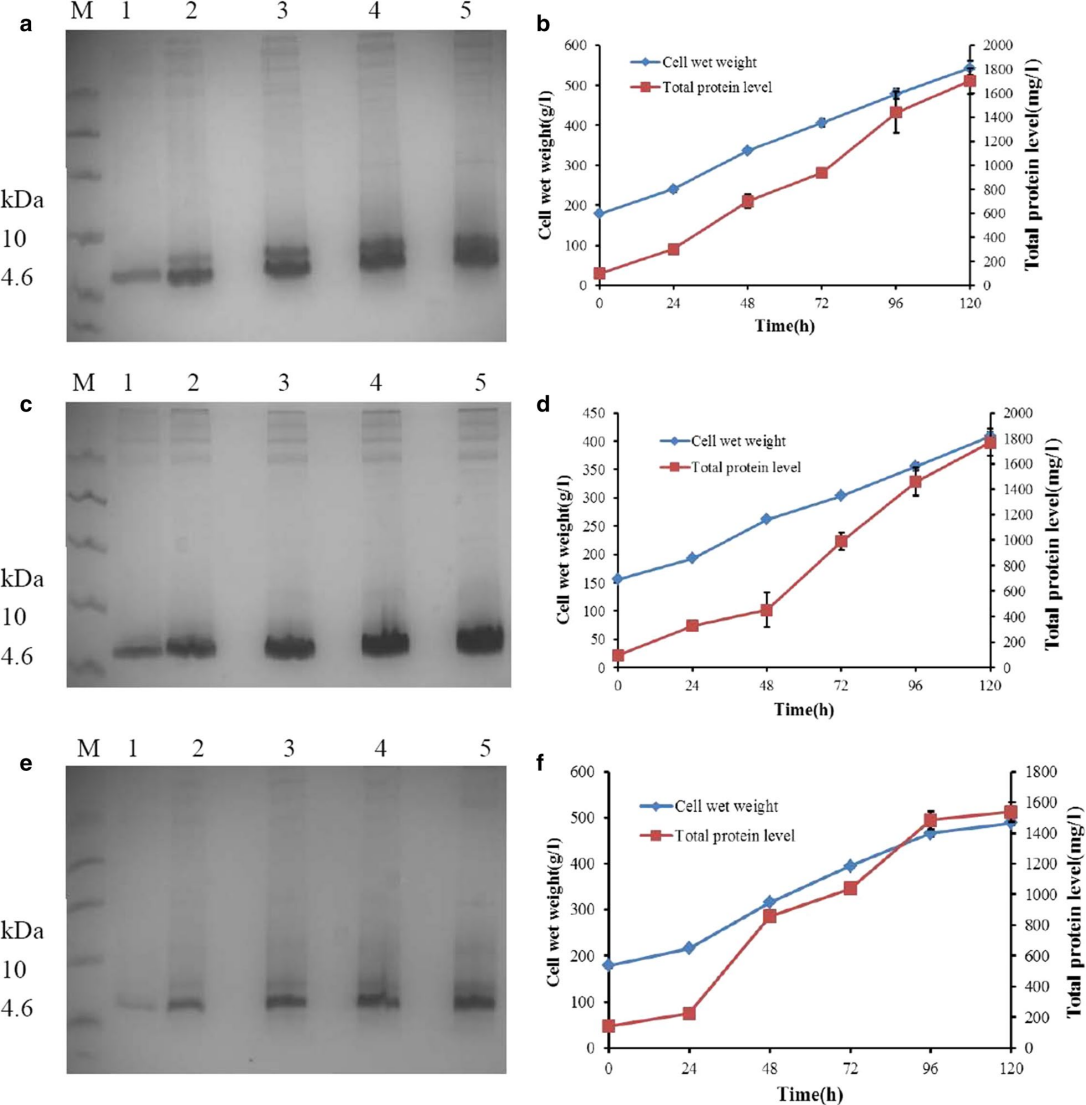
High-density cultivation of H1, H2, H3 in the fermentor level. **a**, **c**, **e** Tricine-SDS-PAGE analysis of H1, H2, H3 fermentation supernatants at different induction times, respectively. Lane 1–5 a total of 5-μl fermentation supernatants taken at 24, 48, 72, 96, 120 h. Lane M 6 μl protein molecular weight marker. **b**, **d**, **f** Time curve of the cell wet weight and total secreted protein levels of H1, H2, H3 during induction (three duplicate observations were made;* bars* represent standard error of mean)

H1, H2, and H3 were cultured and induced in 5-l fermenters. Target peptides in the supernatant were detected after 24 h of induction by Tricine-SDS-PAGE, and the concentration increased with induction time (Fig. 2a, c, e). The cell wet weight of H1, H2, and H3 increased during the induction time and it up to 542.68, 408.97, and 488.98 g/l at 120 h of induction, respectively, and their total protein level reached 1.70, 1.77, and 1.54 g/l, respectively (Fig. 2b, d, f).

### Time-killing curve assay

**Fig. 3:**
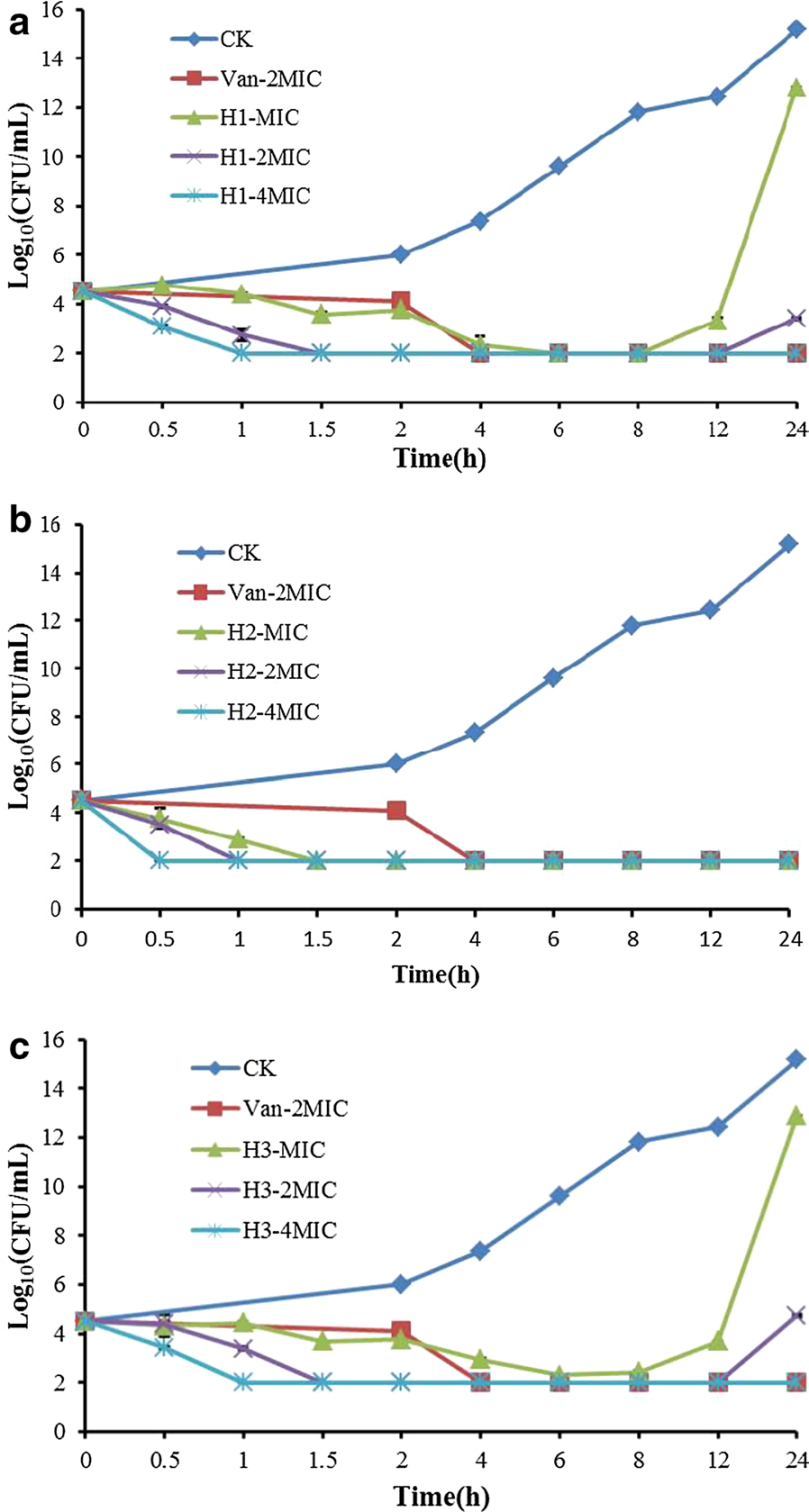
Time-kill curves of H1 (**a**), H2 (**b**) and H3 (**c**). CK: *S. aureus* ATCC43300 (MRSA) were incubated in the presence of medium alone; Van-2MIC: *S. aureus* were incubated in the presence of the vancomycin(Van) at 2× MIC; H1-MIC, H1-2MIC, H1-4MIC, H2-MIC, H2-2MIC, H2-4MIC, H3-MIC, H3-2MIC, H3-4MIC: *S. aureus* were incubated in the presence of H1, H2, H3 at 1× , 2× , 4× MIC, respectively; Three duplicate observation were made; *Bars* represent the standard error of the mean

In vitro time killing curves were performed to evaluate the pharmacodynamic properties and bactericidal ability. In the absence of antimicrobial agent, the bacterial counts (log_10_ CFU/ml) reached to 15.17 for *S. aures* ATCCs43300 at 24 h (Fig. 3). The time killing curves of H1, H2, and H3 had a significant dose-dependent. The bacterial counts decreased to 2 log_10_ CFU/ml (a 99.9% reduction) in 6, 1.5 and 1 h with 1× , 2× , and 4× MIC of H1, respectively. However, after 8 and 12 h, 1× and 2× MIC of H1 had a regrowth and reached to 12.80 and 3.40 log_10_ CFU/ml at 24 h, respectively (Fig. 3a). Meanwhile, the bacterial counts decreased to 2 log_10_ CFU/ml in 1.5, 1, and 0.5 h with 1× , 2× , and 4× MIC of H2, respectively, and maintained to 24 h without regrowth of pathogens (Fig. 3b). The H3 showed a similar trend to H1, but the bacterial counts of 1× MIC of H3 regrowed after 8 h of incubation (Fig. 3c). The efficacy of 1× MIC of H1 and H3 was equivalent to 2× MIC of vancomycin, and all other concentrations of peptides were better than that with vancomycin.

### The postantibiotic effect (PAE) of H1, H2, H3 against *S. aureus*

The PAE of H1 to ATCC43300 was 2.94 h at 2× MIC, which was much longer compared with the 1.43 h for original peptide NZ2114 and 1.72 h for vancomycin at 2× MIC (Table 3). The PAE of H2 and H3 at 1× MIC were 0.63 and 0.55 h, respectively. They had similar values of 1.75 and 1.55 h with NZ2114 and vancomycin at 2× MIC (Table 3).

**Table 3:** The PAE test for H1, H2, H3 against *S. aureus*

Strain	PAE (h)^a^							
	V-2MIC	N-2MIC	H1-MIC	H1-2MIC	H2-MIC	H2-2MIC	H3-MIC	H3-2MIC
*S. aureus* ATCC43300	1.72 ± 0.11	1.43 ± 2.02	0.90 ± 0.04	2.94 ± 0.07	0.63 ± 0.13	1.75 ± 0.04	0.55 ± 0.13	1.55 ± 0.07

*V-2MIC* vancomycin at 2 × MIC, *N-2MIC* NZ2114 at 2 × MIC, *H1-MIC*, *H1-2MIC*, *H2-MIC*, *H2-2MIC*, *H3-MIC*, *H3-2MIC* H1, H2, H3 at 1 × , 2 × MIC, respectively

^a^In vitro observation values are the mean ± SD, n = 3

### Synergism assays of H1, H2, H3 with conventional antibiotics

The synergism effect of H1, H2 and H3 with traditional antibiotics which have different action mechanisms (vancomycin and ampicillin on cell wall, rifampicin on RNA, ciprofloxacin on DNA) was evaluated (Table 4). The MIC values of H1, H2, H3, vancomycin, ampicillin, rifampicin, and ciprofloxacin against ATCC43300 were 0.056, 0.113, 0.056, 0.714, 5.724, 0.019, and 1.510 μM, respectively. When they were used with combination together, almost MIC values were not changed and some MIC values changed to 1/4 or 1/2× MIC. However, the MIC value of ampicillin increased to 2× MIC with H2 and H3. Meanwhile, MICs of H2 and rifampicin increased twice when combined used with ciprofloxacin and H3. The effect of interaction between H1, H2, H3 and antibiotics all showed as indifference with FICI from 1.25 to 3 (Table 4). These results were very different from previous results of original peptide NZ2114 (FICI: 0.125 for vancomycin and ampicillin) (Zhang et al. 2014).

**Table 4:** Combination effects of H1, H2, H3 with traditional antibiotics against *S. aureus*

Combination	Variety	*S. aureus* ATCC43300			
		MIC_a_ (μM)	MIC_c_ (μM)	FIC	FICI
H1-Van	H1	0.056	0.056	1	2
	Van	0.714	0.714	1	
H1-Amp	H1	0.056	0.014	0.25	1.25
	Amp	5.724	5.724	1	
H1-Rif	H1	0.056	0.056	1	2
	Rif	0.019	0.019	1	
H1-Cip	H1	0.056	0.028	0.5	1.5
	Cip	1.510	1.510	1	
H2-Van	H2	0.113	0.113	1	2
	Van	0.714	0.714	1	
H2-Amp	H2	0.113	0.113	1	3
	Amp	5.724	11.450	2	
H2-Rif	H2	0.113	0.056	0.5	1.5
	Rif	0.019	0.019	1	
H2-Cip	H2	0.113	0.227	2	2.5
	Cip	1.510	0.755	0.5	
H3-Van	H3	0.056	0.028	0.5	1.5
	Van	0.714	0.714	1	
H3-Amp	H3	0.056	0.056	1	3
	Amp	5.724	11.450	2	
H3-Rif	H3	0.056	0.056	1	3
	Rif	0.019	0.038	2	
H3-Cip	H3	0.056	0.056	1	1.5
	Cip	1.510	0.755	0.5	

*Van* vancomycin, *Amp* ampicillin, *Rif* rifampicin, *Cip* ciprofloxacin, *H1-Van, H2-Van, H3-Van* H1, H2, H3 in combination with vancomycin, respectively, *H1-Amp, H2-Amp, H3-Amp* H1, H2, H3 in combination with ampicillin, respectively, *H1-Rif, H2-Rif, H3-Rif* H1, H2, H3 in combination with rifampicin, respectively, *H1-Cip, H2-Cip, H3-Cip*, H1, H2, H3 in combination with ciprofloxacin, respectively, *MIC*_*a*_ the MIC of drug alone, *MIC*_*c*_ the MIC of the most effective combination

### Hemolytic assay

Different concentrations of H1, H2, H3, and NZ2114 [0.23–23.09 μM (1–128 μg/ml)] were tested to observe their lysis activity on mice red blood cells (RBCs) in hemolysis assays (Additional file 1: Figure S2). The hemolytic activity of original peptide NZ2114 maintained 1.00% at its concertration from 0.23 to 23.09 μM. Due to the higher charge and hydrophilicity, derived peptides had higher hemolysis compared with NZ2114 but the value was very low in the MICs. H2 and H3 had little or no hemolytic activity at the concentration from 0.23 to 7.27 μM (1–32 μg/ml). H3 had a hemolysis with 6.15% at 14.55 μM (64 μg/ml), while H2 only had 1.51% hemolytic activity at 23.09 μM (128 μg/ml). H1 had higher hemolysis than H2 and H3, which reached 5.99% at 7.27 μM (32 μg/ml).

### CD spectra of H1, H2, H3

**Fig. 4:**
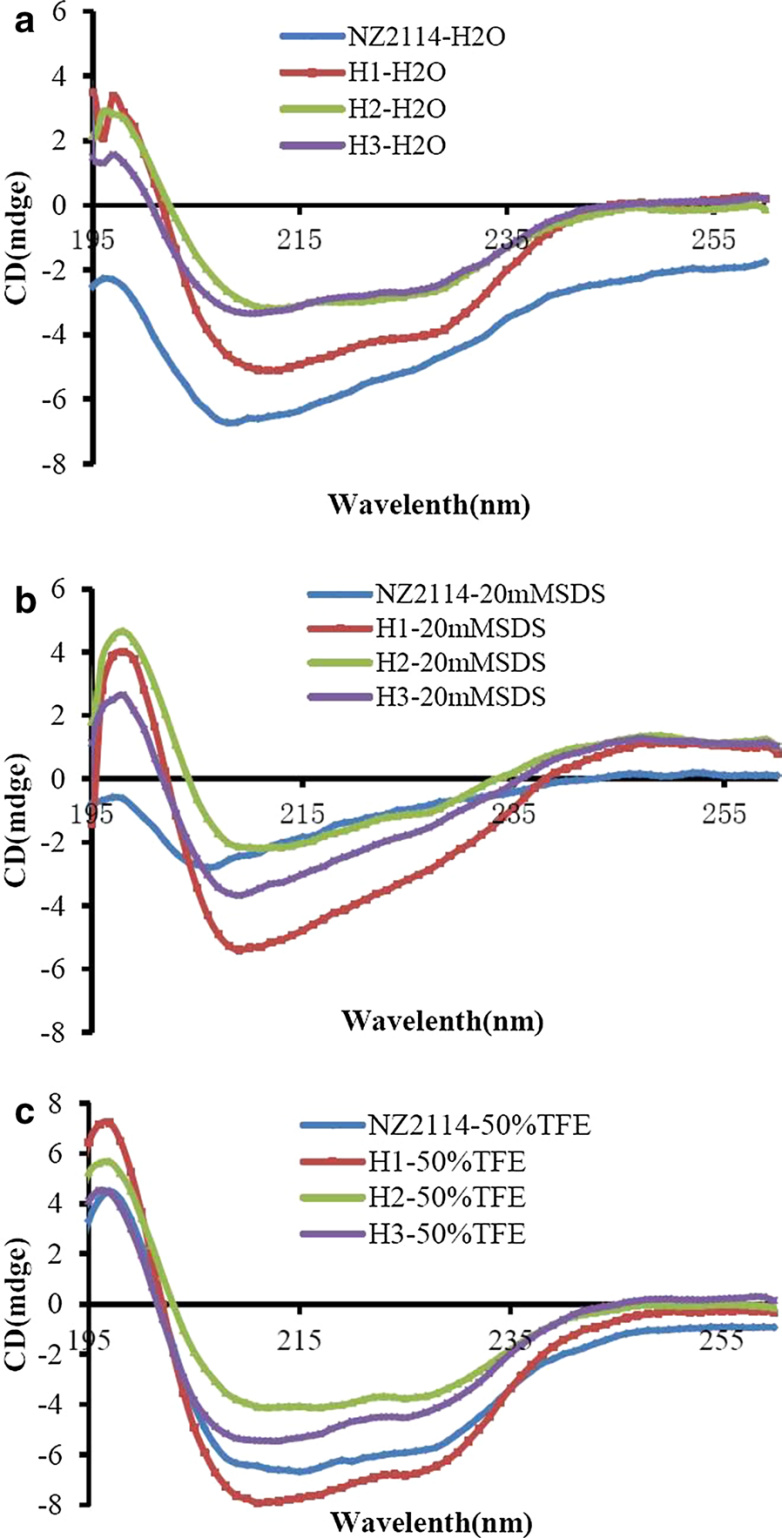
CD spectra of H1, H2, and H3 in different solutions. **a** ddH_2_O; **b** 20 mM SDS; **c** 50% TFE

As shown in Fig. 4 and Table 5, NZ2114 had the high α-helix content of 74.1 and 71.7% in ddH_2_O and 50% TFE, and the content of β-inverse parallel was very low (2.9 and 1.3%). There was lower α-helix (24.8%) and higher antiparallel (18.9%) content in 20 mM SDS. The secondary structures of H1, H2 and H3 were different with NZ2114. The proportion of α-helix was 55.6, 35.6, and 35.1% and the β-inverse parallel was 3.8, 7.7, and 9.8% for H1, H2, and H3 in ddH_2_O, respectively. And similar trends were observed in 20 mM SDS (51.6, 26.8, 35.2% for α-helix; 5.3, 10.3, and 10.5% for β-inverse parallel). However, there was much higher α-helix content (82.9, 52.6, and 63.8% for H1, H2, and H3) and lower β-antiparallel proportion (0.4, 3.0, and 2.5% for H1, H2, and H3) in 50% TFE.

**Table 5:** Percentages of secondary structure of NZ2114, H1, H2 and H3 in different solutions

	NZ2114			H1			H2			H3		
	H_2_O	20 mM SDS	50% TFE	H_2_O	20 mM SDS	50% TFE	H_2_O	20 mM SDS	50% TFE	H_2_O	20 mM SDS	50% TFE
Helix	74.1	24.8	71.7	55.6	51.6	82.9	35.6	26.8	52.6	35.1	35.2	63.8
Antiparallel	2.9	18.9	1.3	3.8	5.3	0.4	7.7	10.3	3.0	9.8	10.5	2.5
Parallel	2.4	9.2	2.9	5.1	5.7	1.4	8.3	9.9	5.5	8.2	8.1	4.0
Beta-turn	13.5	19.0	11.3	14.7	15.8	4.7	16.3	16.5	13.8	17.3	17.7	13.3
Rndm Coil	7.1	28.1	12.8	20.8	21.6	6.6	31.2	36.5	25.1	29.6	28.5	16.4

### Effect of pH, temperature, NaCl concentration and proteinase on the activity of H1, H2, H3

The MIC values of H1 decreased twice (0.057 μM to 0.028 μM) in pH 10 than other pHs and it increased twice (0.057–0.114 μM) in pH 6 than others for H3. Additionally, the MIC of H2 maintained 0.114 μM in all five gradients (Additional file 1: Table S3). Three peptides all showed a high thermal stability, the MIC values of H1 and H3 increased twice only at 100 °C (0.057–0.114 μM), and H2 increased twice in 80 and 100 °C (0.114–0.227 μM) (Additional file 1: Table S4). As shown in Additional file 1: Table S5, the MIC values of H1 (0.057 μM) were not affected in the presence of different NaCl concentrations, but H2 and H3 increased twice at 200 mM (0.114–0.227 μM) and 400 mM (0.057–0.114 μM), respectively. The three peptides showed their own stability to different proteinases (Additional file 1: Table S6). The MIC value of H1 increased twice in pepsin (0.057–0.114 μM), and H2 and H3 increased twice in trypsin (0.227–0.454 μM) and proteinase K (0.057–0.114 μM), respectively.

## Discussion

Currently, 2764 AMPs are registered in the antimicrobial peptide database (APD) (http://aps.unmc.edu/AP/main.php). However, only few AMPs entered into clinical trials. The use of AMPs was primarily hampered by their low oral or intravenous stability, high toxicity, serum binding activity, low activity in physiological condition and so on (Mohammad et al. 2015). Plectasin and its derived peptide NZ2114 had potent activity to *S. aureus*, which are the idea candidates for traditional drugs (Zasloff 2016). However, two histidine residues exist in the sequence of NZ2114, which are uncharged in the physiological condition (Kashiwada et al. 2016). To further improve the antimicrobial activity and properties of NZ2114, new derived peptides are needed.

Although AMPs vary widely in length, structure, and source, they have some important common traits, such as positive charges, presumed to be important for interaction with the negatively charged surface of pathogens, and amphipathicity, which enables better combine with the hydrophilic surface and interact with the hydrophobic part of the microbial membrane (Silva et al. 2014). The arginine and lysine are the key residues for AMPs which are stable charged and hydrophilic in physiological condition. Many studies showed that AMPs having appropriate proportion of arginine and lysine had an improved amphipathicity and higher activity (Veiga et al. 2012; Silva et al. 2014; Gopal et al. 2009; Taniguchi et al. 2014). As results, eight derived peptides (H1–H8) which H16 and H18 were replaced by arginine or lysine were generated.

All derived peptides were tried to express via *P. pastoris* but H4, H5 and H7 were not expressed. The antimicrobial activity of H1, H2, H3, H6 and H8 were assayed and H1, H2, and H3 had higher activity compared with original peptide NZ2114. However, the activity of mutants H6 and H8 which had more net positive charges did not significantly increase (Table 2), which indicated that the electrostatic interaction and cell-penetrating was not the all bactericidal mechanisms of mutants.

Unlike some AMPs with a wide antimicrobial spectrum, H1, H2, and H3 showed a narrow spectrum and they mainly killed the Gram-positive bacterium, such as *S. aureus,* and *Streptococci*, and showed a strong antimicrobial activity (0.007–0.454 μM; Table 2), which was very stronger than the activities of plectasin and NZ2114 (Hara et al. 2008; Zhang et al. 2014). Especially, H1, H2, and H3 showed higher antimicrobial activity against MRSA (*S. aureus* ATCC43300) with MIC of 0.057, 0.114, and 0.057 μM than NZ2114 (0.909 μM) and vancomycin (0.714 μM). Their characteristics of narrow-spectrum antibiotic and low MIC values are very attractive for developing them as candidate agent against MRSA infection.

The lack of economic feasibility to manufacture AMPs at large-scale is another roadblock in the clinical implementation of AMPs (Findlay et al. 2010). Majority of directly expressed AMPs, such as LL-37 (Hong et al. 2007), CecropinAD (Jin et al. 2009), and N2 (Yang et al. 2016), showed unsatisfactory yields. In our previous works, Agplectasin (Mao et al. 2013), NZ2114 (Zhang et al. 2014), and MP1106 (Cao et al. 2015) were expressed in *P. pastoris* X-33 in high level. Similarly, H1, H2, and H3 were expressed in *P. pastoris* X-33 with high yields, their total protein level in 5-l fermentation reached 1.70, 1.77, and 1.54 g/l, respectively (Fig. 2b, d, f). However, because the fermentation was performed in summer, the temperature cannot be controlled at 29 °C as previous operation. To maintain the dissolved oxygen content, we had to reduce the flow rate, which lead to the partial losing of yield. If the induction temperature can maintain at 29 °C, at which this key temperature is very important for its high expression in yeast (Li et al. 2001, 2007). the production of H1, H2, H3 may be further improved like their original peptide NZ2114 (2390 mg/l in 29 °C and 2310 mg/l in 25 °C) (Zhang et al. 2014).

The PAE is a very important pharmacodynamics parameter in choosing of antibiotic dosage regimens in clinical use (Pankuch and Appelbaum 2009a). Obviously observed, the PAEs of H1, H2, and H3 increased with the concentration from 1× MIC to 2× MIC (Table 3). They showed similar values to vancomycin (2× MIC: 1.72 h) and NZ2114 (2× MIC: 1.43 h). H1 also had comparative value (2× MIC: 2.94 h) to some conventional antibiotics, for instance, daptomycin (2.0 h), tigecycline (3.2 h), and arbekacin (3.0–3.2 h), respectively (Pankuch and Appelbaum 2009b; Pankuch et al. 2003; Watanabe et al. 1997). Their appropriate PAE is critical to lengthen the interval of administration, reduce the daily dosages, and thus potentially reduce potential drug resistance.

To combat antibiotic resistance, combination antibiotic therapy is practiced in the clinical use due to its advantages such as wider coverage, higher activity, bactericidal synergy and the inhibition on toxin production (Leibovici et al. 2010; Müller et al. 2013). Vancomycin, one of the most effective antibiotics against MRSA, is often combined used with rifampicin, gentamicin, dalfopristin, and β-lactams to slow the development of resistance and enhance the antibacterial activity. The synergistic effect of H1, H2, and H3 is very different from their parent peptide NZ2114 (Zhang et al. 2014). The FICI of NZ2114 combined with ampicillin, and vancomycin to *S. aureus* ATCC43300 was 0.125, showing additivity effect (Zhang et al. 2014). However, the indifference effects (1.25 ≤ FICI ≤ 3) were observed for all combinations between H1, H2, H3 and four traditional antibiotics to *S. aureus* ATCC43300 (Table 4), which might result from the changed antimicrobial property and mechanism and its details should be studied in our next research.

The plectasin and NZ2114 showed no hemolysis in rabbit and human RBCs (Yang et al. 2011; Zhang et al. 2014). In this work, H1, H2, and H3 showed low hemolysis in mice RBCs. Although the values were higher than NZ2114 due to the high charge and hydrophilicity, they were still very low in the range of MICs. In addition, NZ2114 showed the highest activity in pH value of 8.0 and above 80% of initial activity was retained over a range of temperatures from 20 to 80 °C but maintained 20% activity at 100 °C (Zhang et al. 2014). Owing to contribution from the three pairs of disulfide bond into the stability of structure, H1, H2, and H3 all had high stability with minor differences. H1 was not sensitive to NaCl concentration, but sensitive to alkaline and high temperature environments. H2 was sensitive to high temperature and NaCl concentration, but not sensitive to different pHs environment. H3 was sensitive to high temperature and high NaCl concentration. Generally, no toxicity to erythrocytes and high stability of pH, temperature, proteases, and saline ions of H1, H2 and H3 meet the key requirements of new antimicrobial agents.

In summary, series of novel AMPs were designed and successfully expressed in *P. pastoris*. Among them, H1, H2, and H3 had high yields (1.70, 1.77 and 1.54 g/l) in 5-l fermentor level. H1, H2, and H3 also showed strong antimicrobial activity against *S. aureus*. They killed MRSA strain ATCC43300 in a short time with low concentrations and had long post antibiotic effect. Meanwhile, H1, H2, and H3 exhibited indifference effects when they were combined with conventional antibiotics. Furthermore, they had low toxicity to mice erythrocytes and high stability. All results indicate that H1, H2, and H3 have potential as candidates for the therapeutic agents with the better properties than their native peptide NZ2114.

## Additional file

**Additional file 1.** Additional tables and figures.

## Abbreviations

MIC: minimal inhibitory concentration
RBCs: red blood cells
AMP: anitimicrobial peptide
APD: antimicrobial peptide database
