# Supplementary material for: Design and pharmacodynamics of recombinant NZ2114 histidine mutants with improved activity against methicillin-resistant Staphylococcus aureus
Source: AMB Express. 2017 Feb 22;7:46. doi: 10.1186/s13568-017-0345-x (PMC5321639; doi:10.1186/s13568-017-0345-x)
Supplement: Supplementary file 1 — Additional file 1. Additional tables and figures. [file 13568_2017_345_MOESM1_ESM.pdf]

AMB Express

Design and pharmacodynamics of recombinant NZ2114 histidine mutants with improved activity against methicillin-resistant *Staphylococcus aureus*

Huixian Chen[1, 2, 3], Ruoyu Mao[2, 3*], Da Teng[2, 3], Xiumin Wang[2, 3], Ya Hao[2, 3], Zhanzhan Li[2, 3], Xingjun Feng[1*], Jianhua Wang [2, 3*]

1 Institute of Animal Nutrition, Northeast Agricultural University, Harbin 150030, China.

2 Key Laboratory of Feed Biotechnology, Ministry of Agriculture, Beijing 100081, China.

3 Gene Engineering Laboratory, Feed Research Institute, Chinese Academy of Agricultural Sciences, Beijing 100081, China.

* Correspondence should be addressed to:

1) Prof., Ph.D., and PI. Jianhua Wang:

E-mail address: wangjianhua@caas.cn; 2681298635@qq.com

Phone: 0086-10-82106081

2) Dr., Professor Xingjun Feng

E-mail address: fengxingjun2008@163.com

Phone: 0086-451-55191395

3) Dr., A/S Prof, Ruoyu Mao

E-mail address: maoruoyu@caas.cn

Phone: 0086-10-82106081

Supplementary information

Supplementary Table S1 Strains used in the antimicrobial activity assays.

Supplementary Table S2 Nucleotide sequence of H1-H8

Supplementary Table S3 MIC values of H1, H2, H3 in different pHs.

Supplementary Table S4 MIC values of H1, H2, H3 in different temperatures.

Supplementary Table S5 MIC values of H1, H2, H3 in different NaCl concentrations.

Supplementary Table S6 MIC values of H1, H2, H3 in different proteinases.

Supplementary Figure S1 Tricine-SDS–PAGE of the supernatant of H1~H8 expressed in 48-well plates.

Supplementary Figure S2 Hemolytic activity of H1, H2, H3, and NZ2114 against mice erythrocytes.

Supplementary Table S1 Strains used in the antimicrobial activity assays

Strains	Source
Gram-positive bacteria	
Staphylococcus aureus ATCC25923	CVCC[a]
S. aureus ATCC43300 (MRSA)	Yansheng (Shanghai, China)
S. aureus ATCC6538	CVCC
Streptococcus suis CVCC3309	CVCC
S. suis CVCC3928	CVCC
S. suis CVCC606	CVCC
S. pneumonia CVCC1.8722	CVCC
S. pneumonia CVCC2350	CVCC
Gram-negative bacteria	
Salmonella enteritidis CMCC50336	CMCC[b]
S. typhimurium ATCC14028	CVCC
S. choleraesuis CVCC503	CVCC
S. pullorum CVCC1789	CVCC
Escherichia coli CVCC195	CVCC
E. coli CICC21530	CICC[c]

[a] China Institute of Veterinary Drug Control

[b] China General Microbiological Culture Collection Center

[c] China Center of Industrial Culture Collection

Supplementary Table S2 Nucleotide sequence of H1-H8

Name	Sequence
NZ16K (H1)	GGTTTTGGTTGTAACGGTCCATGGAACGAAGATGATTTGAGATGTAAGAACCATTGTAAGTCTATTAAGGGTTACAAGGGTGGTTACTGTGCTAAGGGTGGTTTTGTTTGTAAGTGTTAC
NZ16R (H2)	GGTTTTGGTTGTAACGGTCCATGGAACGAAGATGATTTGAGATGTAGAAACCATTGTAAGTCTATTAAGGGTTACAAGGGTGGTTACTGTGCTAAGGGTGGTTTTGTTTGTAAGTGTTAC
NZ18K (H3)	GGTTTTGGTTGTAACGGTCCATGGAACGAAGATGATTTGAGATGTCATAACAAGTGTAAGTCTATTAAGGGTTACAAGGGTGGTTACTGTGCTAAGGGTGGTTTTGTTTGTAAGTGTTAC
NZ18R (H4)	GGTTTTGGTTGTAACGGTCCATGGAACGAAGATGATTTGAGATGTCATAACAGATGTAAGTCTATTAAGGGTTACAAGGGTGGTTACTGTGCTAAGGGTGGTTTTGTTTGTAAGTGTTAC
NZ16K18K (H5)	GGTTTTGGTTGTAACGGTCCATGGAACGAAGATGATTTGAGATGTAAGAACAAGTGTAAGTCTATTAAGGGTTACAAGGGTGGTTACTGTGCTAAGGGTGGTTTTGTTTGTAAGTGTTAC
NZ16K18R (H6)	GGTTTTGGTTGTAACGGTCCATGGAACGAAGATGATTTGAGATGTAAGAACAGATGTAAGTCTATTAAGGGTTACAAGGGTGGTTACTGTGCTAAGGGTGGTTTTGTTTGTAAGTGTTAC
NZ16R18K (H7)	GGTTTTGGTTGTAACGGTCCATGGAACGAAGATGATTTGAGATGTAGAAACAAGTGTAAGTCTATTAAGGGTTACAAGGGTGGTTACTGTGCTAAGGGTGGTTTTGTTTGTAAGTGTTAC
NZ16R18R (H8)	GGTTTTGGTTGTAACGGTCCATGGAACGAAGATGATTTGAGATGTAGAAACAGATGTAAGTCTATTAAGGGTTACAAGGGTGGTTACTGTGCTAAGGGTGGTTTTGTTTGTAAGTGTTAC

Supplementary Table S3 MIC values of H1, H2, H3 in different pHs

	MIC(μM)				
	pH2	pH4	pH6	pH8	pH10
H1	0.057	0.057	0.057	0.057	0.028
H2	0.114	0.114	0.114	0.114	0.114
H3	0.057	0.057	0.114	0.057	0.057

Supplementary Table S4 MIC values of H1, H2, H3 in different temperatures

	MIC(μM)					
	4°C	20°C	40°C	60°C	80°C	100°C
H1	0.057	0.057	0.057	0.057	0.057	0.114
H2	0.114	0.114	0.114	0.114	0.227	0.227
H3	0.057	0.057	0.057	0.057	0.057	0.114

Supplementary Table S5 MIC values of H1, H2, H3 in different NaCl concentrations

	MIC(μM)					
	50mM	100mM	200mM	300mM	400mM	500mM
H1	0.057	0.057	0.057	0.057	0.057	0.057
H2	0.114	0.114	0.227	0.227	0.227	0.227
H3	0.057	0.057	0.057	0.057	0.114	0.114

Supplementary Table S6 MIC values of H1, H2, H3 in different proteinases

	MIC(μM)		
	Trypsin	Pepsin	Proteinase K
H1	0.057	0.027	0.057
H2	0.454	0.227	0.227
H3	0.057	0.057	0.114

Supplementary Figure S1

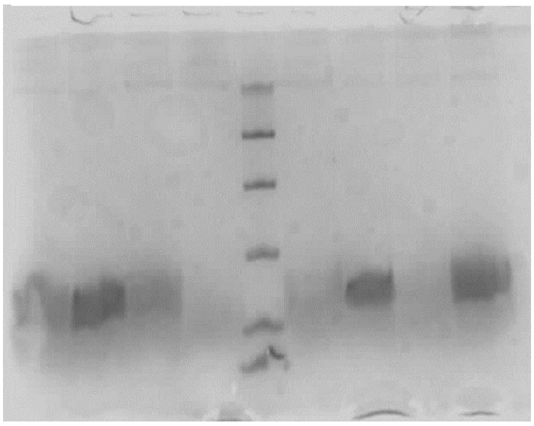

Fig. S1 Tricine-SDS–PAGE of the supernatant of H1~H8 expressed in 48-well plates. Lane M 6 μl of protein molecular weight marker; Lane 1~8 20 μl supernatant of H1~H8 expressed in 48-well plates.

Supplementary Figure S2

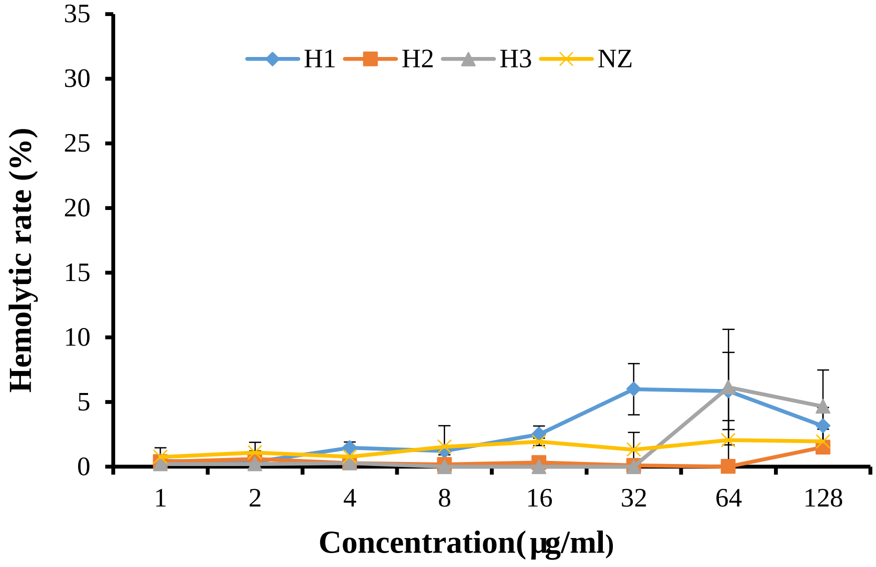

Fig. S2 Hemolytic activity of H1, H2, H3, and NZ2114 against mice erythrocytes. Three duplicate observations were made; Bars represent the standard error of the mean.
